# Accuracy of Infrared Thermography in Diagnosing Breast Cancer-Related Lymphedema

**DOI:** 10.3390/jcm13206054

**Published:** 2024-10-11

**Authors:** Vanessa Maria da Silva Alves Gomes, Marcos Leal Brioschi, Ana Rafaela Cardozo da Silva, Naiany Tenório, Laura Raynelle Patriota Oliveira, Ana Claúdia Souza da Silva, Juliana Netto Maia, Diego Dantas

**Affiliations:** 1 Departamento de Fisioterapia, Centro de Ciências da Saúde, Universidade Federal de Pernambuco, Av. Jornalista Aníbal Fernandes, 173, Cidade Universitária, Sala 2, 1º andar, Recife 50740-560, PE, Brazil; vanessaalvesfta@gmail.com (V.M.d.S.A.G.); rafaela.cardozo@ufpe.br (A.R.C.d.S.); n.tenorio.j@gmail.com (N.T.); laurapatriotaa@gmail.com (L.R.P.O.); claudia.souzasilva@ufpe.br (A.C.S.d.S.); juliana.netto@ufpe.br (J.N.M.); 2 Medical Thermology and Thermography Specialization, Hospital das Clínicas da Faculdade de Medicina da Universidade de São Paulo, São Paulo 05403-010, SP, Brazil; marcosbrioschi@gmail.com

**Keywords:** breast cancer, lymphedema, thermography

## Abstract

**Background/Objectives:** Infrared thermography (IRT) is an imaging technique used in clinical practice to detect changes in skin temperature caused by several dysfunctions, including breast cancer-related lymphedema (BCRL). Thus, the present study aimed to assess the reproducibility and accuracy of IRT in diagnosing BCRL. **Methods:** This cross-sectional study included participants who underwent a unilateral mastectomy and used indirect volumetry for lymphedema detection. IRT analysis was recorded in four positions, analyzing maximum, mean, and minimum temperatures, as well as the temperature differences between the upper limbs. The analysis encompassed reliability, agreement, accuracy, and the establishment of cut-off points for sensitivity and specificity. A total of 88 upper limbs were included; 176 thermograms were captured, and 1056 regions of interest were analyzed. **Results:** IRT presented excellent intra- and inter-rater reproducibility and reliability with excellent intraclass correlation coefficient values (0.99 to 1.00). In addition, this assessment reached a sensitivity of 85% and a specificity of 56%; the cut-off point considered a temperature difference of −0.45 °C. **Conclusions:** IRT was a reliable and reproducible assessment, and the temperature difference between the upper limbs evidenced moderate accuracy. Thus, IRT is recommended as a complementary technique for detecting BCRL.

## 1. Introduction

Infrared thermography (IRT) is a noninvasive imaging technique that captures infrared radiation emitted by the skin, providing real-time thermal patterns that reflect tissue dysfunctions that affect thermoregulation. This analysis is of interest in healthcare due to its potential for diagnostic and monitoring purposes in clinical practice [1], such as in breast cancer-related lymphedema (BCRL) [2].

BCRL is a chronic condition marked by metabolic and vascular alterations that cause lipid accumulation and fibrosis. Common signs and symptoms are swelling, heaviness, pain, and paresthesia [2]. These alterations affect skin temperature, making IRT a promising technique for the early diagnosis, evaluation, and monitoring of BCRL [3,4]. The diagnosis of lymphedema usually involves a combination of patient history and complementary assessments.

Despite the existence of several techniques, such as electrical impedance spectroscopy, tomography, and lymphoscintigraphy, their use in clinical practice is challenging due to high costs and limited accessibility [5,6,7]. Thus, indirect volumetry from circumference measurements of the upper limb (UL) remains the most used technique in clinical practice due to its good accessibility and reproducibility. However, the accuracy of the diagnosis is limited by its rater-dependent nature, lack of measurement standardization, and inability to detect fluid distribution variations that do not change the UL volume or size [8].

This challenge highlights the need for new, low-cost, non-invasive, accurate, and standardized techniques to ensure the early diagnosis and treatment of BCRL, improving clinical outcomes and reducing costs [9]. Therefore, IRT represents a promising complementary assessment for BCRL diagnosis, particularly as an IRT point-of-care technique. This non-invasive technique detects thermal variations in the skin that indicate changes in circulation and fluid distribution in subclinical stages [10]. Additionally, IRT facilitates real-time monitoring of skin temperature, optimizing assessment time and providing insights into tissue metabolic changes in BCRL [3,10].

Although previous studies investigated IRT in BCRL, they did not assess the reliability and reproducibility of this technique. Technical factors, the selection of regions of interest (ROI), analysis methods, and environmental conditions are parameters that impact results, influencing the diagnostic accuracy of the technique. Therefore, the present study aimed to assess the reproducibility and accuracy of IRT in BCRL diagnosis. By focusing on temperature specifications and defining diagnostic cutoff points, this research aims to provide a reliable framework for integrating IRT into clinical practice as a complementary tool for the early detection of lymphedema.

## 2. Materials and Methods

### 2.1. Study Design

This prospective cross-sectional study aimed to assess the reproducibility and accuracy of infrared thermography (IRT) in diagnosing breast cancer-related lymphedema (BCRL). The study was approved by the research ethics committee (CAAE: 57624121.0.0000.5208), and all the participants provided informed consent following the Declaration of Helsinki.

### 2.2. Participants

The eligible participants were women breast cancer survivors, aged between 40 and 70 years, with a history of a unilateral mastectomy. Exclusion criteria included a history of bilateral breast cancer, undergoing chemotherapy or radiotherapy treatment, primary lymphedema, edema associated with other causes (e.g., rheumatological, renal, neurological, orthopedic problems, or previous vascular diseases), erysipelas, intertrigo, ulcers, psoriasis, atopic dermatitis (eczema), seborrheic dermatitis, cellulitis, Hansen’s disease, vitiligo, scabies, herpes zoster, inflammatory acne, folliculitis, and other pre-existing skin conditions that could affect skin thermoregulation.

### 2.3. Recruitment Process

The participants were recruited via social media announcements, clinic partnerships, and referral hospitals. Interested participants underwent an initial screening at the institution’s Physiotherapy Laboratory. A dermatologist then reviewed each participant, verifying eligibility based on predefined criteria. A total of 120 participants were approached, and 44 met the eligibility criteria. Those eligible for inclusion criteria and willing to participate signed the informed consent form in accordance with Resolution 466/12 of the National Health Council.

### 2.4. Clarification of Group Allocation

The participants were allocated to two groups: those with lymphedema (upper limb lymphedema—ULWL) and those without (upper limb control—ULC). Allocation was determined based on volumetric assessments, not randomized. This methodology helped minimize bias by ensuring objective differentiation between those with and without lymphedema based on standardized diagnostic criteria.

### 2.5. Data Collection and Assessor Training

The data collection process involved two evaluators who underwent prior training for all stages of data collection. Initially, participants filled out a clinical form containing information about age, body mass index, dominant side (right or left), upper limb ipsilateral to mastectomy (right or left), time since cancer diagnosis and time since mastectomy, and others. treatments (chemotherapy, radiotherapy and hormonal therapy). Subsequently, indirect volumetric measurements were carried out to investigate the presence of lymphedema in the participants’ upper limbs and then the thermographic image of the upper limbs was recorded, followed by the analysis of the thermograms.

### 2.6. Volumetry for Lymphedema Detection

Lymphedema was evaluated using indirect volumetry based on the truncated cone formula, a method endorsed by the International Society of Lymphology for its high diagnostic accuracy and reproducibility [6,11]. This technique calculates upper limb (UL) volumes using circumference measurements at five fixed points, with the styloid process of the ulna serving as a reference. Measurements were taken with participants standing in a standardized position: shoulder abducted at 90 degrees, elbow extended, wrist in ulnar deviation, and hand touching the wall [12]. Lymphedema was diagnosed when the volume difference between the ipsilateral and contralateral ULs was ≥200 mL or the volume ratio exceeded 1.04 [11].

### 2.7. Imaging Methods

#### 2.7.1. Pre-Examination Recommendations

Prior to image acquisition, the participants were instructed to fast for up to three hours and avoid using creams or perfumes on their skin, consuming biostimulant substances, and engaging in vigorous exercise two hours before the examination. Menstruating participants were instructed to schedule the examination outside of the luteal phase to minimize interference with IRT temperature measurements due to thermal variations throughout the menstrual cycle. Core body temperature may increase during the post-ovulatory luteal phase due to higher progesterone levels, compared to the pre-ovulatory follicular phase, where progesterone levels are lower.

#### 2.7.2. Image Acquisition

**Figure 1:**
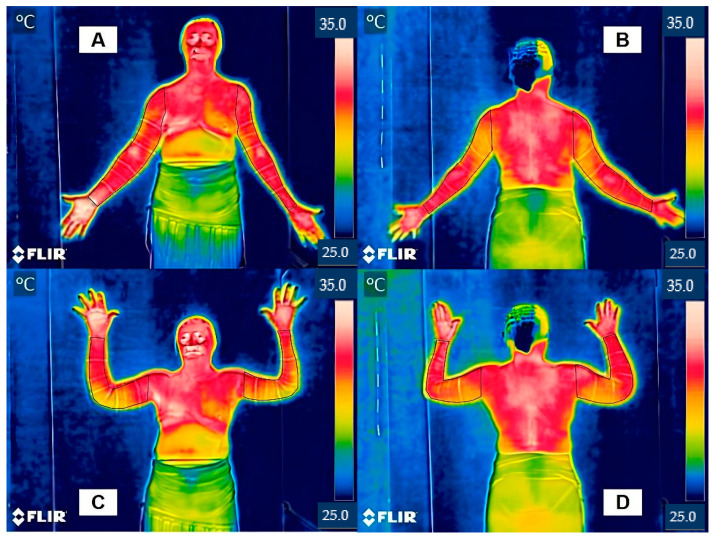
Representation of the region of interest in different positions for infrared thermography in the diagnosis of breast cancer-related lymphedema. Subfigures show the positions for capturing the infrared thermography images: (**A**) anterior anatomical position (frontal area of upper limbs); (**B**) posterior anatomical position (posterior area of upper limbs); (**C**) anterior position with arms abduction (anterior lateral region); and (**D**) posterior position with arms abduction (posterior lateral region).

The IRT images were acquired in a controlled environment (i.e., windowless room with a temperature of 23 °C and a relative humidity of 55%) regulated by a digital weather station, and away from heat generated by electronic devices [13]. Upon entering the room, the participants undressed and exposed their upper limbs, chest, and abdomen for 15 min to allow thermal equilibration with the room temperature [14]. During imaging, the participants stood barefoot on a rubber mat positioned in four standardized postures to ensure comprehensive thermal analysis (Figure 1):

(A) Anterior anatomical position (AAP): anterior view; the participant is in an orthostatic position with their ULs aligned alongside the trunk and their wrist in a neutral position with finger extension, and their forearm in supination;
(B) Posterior anatomical position (PAP): posterior view; the participant is in an orthostatic position with their ULs aligned alongside the trunk, their wrist in a neutral position with finger extension, and their forearm in supination;
(C) Anterior position with arms abduction (APWA): anterior view; the participant is in an orthostatic position with their shoulders flexed, abducted, and externally rotated, their elbows flexed, neutral wrists, and their forearms in pronation;
(D) Posterior position with arms abduction (PPWA): posterior view; the participant is in an orthostatic position with their shoulders flexed, abducted, and externally rotated, their elbows flexed, neutral wrists, and their forearms in pronation.

During image capture, the camera was placed on a tripod at a height of 75 cm from the ground and remained operational during the acclimatization period to calibrate its sensor. Participants were positioned 1 m away from the camera, allowing a clear visualization of the ROI. The images were saved with codes to maintain blinding during the IRF analysis. The data storage and protection process followed the recommendations of the Brazilian General Data Protection Law, Law No. 13,709, of 14 August 2018.

#### 2.7.3. Camera Type and Calibration

Skin temperature was obtained using a portable multispectral camera (TermoCam medical standard C5, FLIR Systems^®^) with the following specifications: a measurement range of −20 to 50 °C, an infrared spectral band of 8 to 14 µm, a thermal imaging resolution of 320 × 240 pixels, an emissivity (ε = 0.98), and a focal length of 1 m.

The device was calibrated one week before the start of collection using a blackbody source with an uncertainty of less than 0.1 °C (95% confidence level) and stability better than ±0.002 °C. Calibration focused on the human face, requiring the operator to frame the facial image within the target plane for accurate data collection [15]. The calibrations were performed by a laboratory competent in radiation thermometric calibrations, traceable to the international measurement standard. For this purpose, the camera calibration was performed using radiation sources that were traceable to the National Standards at RISE, to the Research Institutes of Sweden, and to NIST, the National Institute of Standards and Technology (USA). The blackbody source had a sufficient radiance temperature range and control span for laboratory testing according to this standard. The blackbody source had a known emissivity greater than 0.995. The aperture diameter of the blackbody source was sufficiently large so that the temperature measurement of the thermal imager was not affected by it and to allow a clear identification of the color change in the functional plane. Furthermore, studies have shown that portable cameras have sensitivity and specifications, with performance comparable to more sophisticated camera models, for the medical diagnosis of various conditions [1].

#### 2.7.4. Thermogram Analysis

The IRT images were analyzed using Thermofy^®^ software version 1.2.1 (Infraredmed, Brazil), with a black screen in the background serving as a temperature reference. Regions of interest (ROI) were delineated by polygons delimiting the UL from the styloid process of the ulna to the acromial region. The evaluators established these polygons independently to include as much of the body segment as possible in the analysis. A temperature range between 25 °C and 35 °C was established to delineate the ROIs in the images. Each IRT image was analyzed three times to ensure data reproducibility: twice by the same evaluator, with a seven-day interval between analyses, and once by a second independent evaluator [16].

The evaluators, who had previous experience, training, and calibration using Thermofy software, were blinded to the allocation of participants into the two groups: upper limb with lymphedema (ULWL) and control upper limb without lymphedema (ULC). The defined ROIs were measured for maximum, mean, and minimum temperatures in Celsius (°C) [17]. Figure 1 illustrates the ROI delineation process.

### 2.8. Data Analysis and Interpretation

Data were analyzed using SPSS (version 20.0) and expressed as the mean and standard deviation for quantitative variables or the absolute and relative frequency for qualitative variables. A prevalence of 40% for BCRL was considered for the sample size calculation [18], with a minimum specificity of 70%, statistical power of 80%, and error of 0.048. The estimated minimum sample size was 52 ULs: 21 from the ULWL and 31 from the ULC group [19]. A post hoc analysis performed using GPower (version 3.1) (Universität Düsseldorf) indicated that the included sample of 88 ULs (26 with and 62 without lymphedema) achieved a statistical power of 89% to detect differences in maximum temperature between the ULs, with a mean and standard deviation (SD) of 31.92 (1.74) for ULWL and 32.447 (1.35) for ULC.

Intra- and inter-rater reproducibility was assessed by calculating the intraclass correlation coefficient (ICC) and comparing ipsi- and contralateral ULs in the groups. The analysis was conducted using a 95% confidence interval and based on the two-way mixed effects, single measure, and absolute agreement. The ICC values were interpreted as having good (0.75 to 0.90) and excellent (>0.90) reliability [16]. The standard error of measurement (SEM) and minimal detectable change (MDC) were calculated to investigate the expected variability in repeated measurement results and to assess the accuracy of the measurement, respectively.

The receiver-operating characteristic (ROC) curve was used to assess the accuracy of IRT in BCRL diagnosis. The analysis of the area under the curve (AUC) provided a quantitative measure of the ability of the image examination to distinguish between health and disease conditions. The AUC values were classified as a random test (0.5), low accuracy (>0.5 to 0.7), moderate accuracy (0.7 to 0.9), high accuracy (>0.9 to <1.0), and a perfect test (1.0). The ideal cut-off point was calculated using the Youden index, defined as (sensitivity + specificity −1). This method identifies the point that maximizes the effectiveness of the test in distinguishing between ULWL and ULC groups. Additionally, the evaluation included the analysis of false positives, false negatives, true positives, and true negatives. The application of the index resulted in cut-off points that were sensitive and specific, facilitating the clinical interpretation of the results. Reproducibility analyses were conducted to evaluate the intra-rater variation of the same participant at two time points with a seven-day interval [20].

Furthermore, the accuracy of the IRT images was investigated considering the temperature difference (ΔT) between the ULs. The analysis focused on how the cutoff points for the upper limb skin temperature changes described in the literature influence the diagnosis of BCRL, with temperature differences of 0.3 °C (suggestive of abnormality), 0.6 °C (strongly suggestive), and 1.0 °C (significant abnormality) [21].

## 3. Results

**Figure 2:**
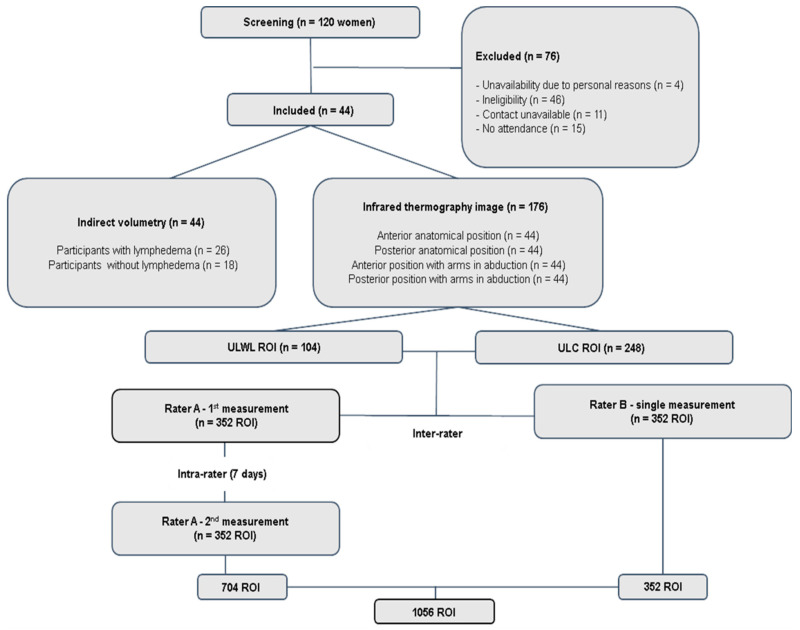
Flowchart of the screening and data collection process. This flowchart outlines participant selection, delineates inclusion and exclusion criteria, and classifies the participants as upper limbs with lymphedema (ULWL) or upper limbs control (ULC).

The sample included 88 ULs: 26 from ULWL and 62 in the ULC group. A total of 176 IRT images were acquired, resulting in 352 ROIs: 104 for the ULWL and 248 for the ULC group. Considering the intra- and inter-rater analyses, 1056 ROIs were analyzed (Figure 2). The sociodemographic and clinical characterization of the participants are presented in Table 1. Most of the BCRL cases in this study were considered chronic.

The intra- and inter-rater reliability of thermograms analyses was excellent in all the positions, with the ICC ranging from 0.97 to 1.00. In the inter-rater assessment, SEM ranged from 0.00 to 0.07 °C for both groups, while the MDC ranged between 0.00 and 0.22 °C for ULWL and from 0.00 to 0.07 °C for ULC. In the intra-rater analysis, SEM ranged from 0.00 to 0.11 °C for ULWL and from 0.00 to 0.07 °C for ULC. The MDC ranged from 0.00 to 0.21 °C for ULWL and from 0.00 to 0.07 °C for ULC. For all the positions, the SEM and MDC values were close to zero, indicating excellent reproducibility and measurement accuracy, respectively. The reliability of the IRT measurements illustrating the maximum, mean, and minimum temperatures in the ULWL and ULC groups are presented in Table 2.

The accuracy indices of infrared thermography (IRT) for the skin temperature of the upper limbs demonstrated low accuracy (0.546 to 0.632) with wide variability in AUC values (from 0.414 to 0.559), sensitivity (0.500 to 0.692), and specificity (0.565 to 0.661) for the evaluated positions. This suggests that analyzing the skin temperature of the upper limb alone is not the best method for diagnosing lymphedema (Table 3).

**Figure 3:**
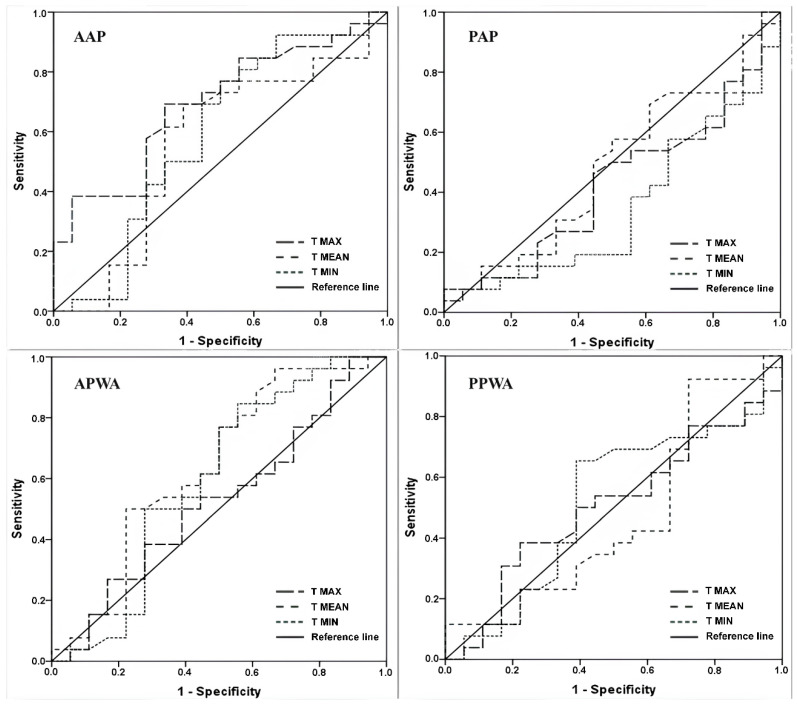
Receiver-operating characteristic curves compare skin temperatures in upper limbs (maximum, mean, and minimum), differentiating between upper limbs with lymphedema and those without lymphedema in all positions.

**Figure 4:**
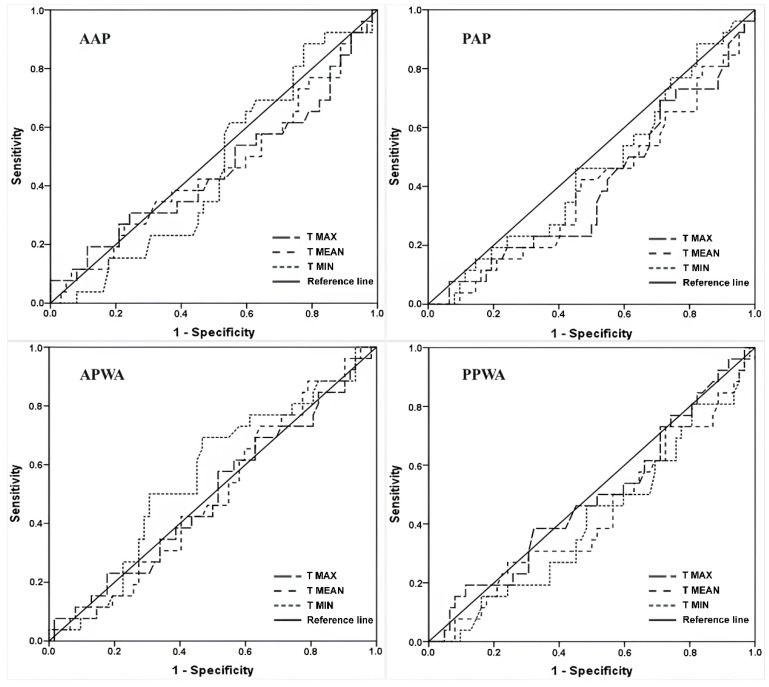
Receiver-operating characteristic curves of maximum, mean, and minimum skin temperatures for delta values in each position.

The accuracy indices of infrared thermography (IRT) for the ΔT of the upper limbs (ULs) are presented in Table 4. The AUC ranged from 0.518 to 0.700, with the maximum temperature in the anterior anatomical position (AAP) being the best compared to other positions, showing moderate accuracy and a cut-off point of −4.5 °C (AUC = 0.700, sensitivity = 0.846, and specificity = 0.556). The anterior position with arms abduction (APWA) presented the highest AUC for mean and minimum temperatures, although it demonstrated low accuracy, with cut-off points of −1.2 °C (AUC = 0.637, sensitivity = 0.769, and specificity = 0.556) and −2.5 °C (AUC = 0.595, sensitivity = 0.885, and specificity = 0.722), respectively. Figure 3 and Figure 4 illustrate the graphical distribution using the ROC curve.

Table 5 presents the sensitivity and specificity of IRT in detecting lymphedema, using cut-off points established in the literature (0.3 °C, 0.6 °C, and 1.0 °C), based on ΔT values measured in the AAP and APWA positions. It is observed that as the cut-off point increases, diagnostic accuracy improves, yielding higher sensitivity and specificity values. These findings highlight the efficacy of IRT in identifying lymphedema, validating its use as a screening tool and establishing optimal parameters for future clinical applications.

## 4. Discussion

Skin temperatures were evaluated in the ULWL and ULC groups to investigate IRT as a technique to detect BCRL. IRT emerged as a reliable and reproducible instrument suitable for the clinical management of BCRL. Additionally, the maximum ΔT value observed in the AAP was the most suitable parameter for BCRL evaluation.

In this study, the IRT of the ULs was performed in four positions, revealing that AAP and APWA exhibited higher sensitivity and specificity values; the former was the most suitable for participants with BCRL because it is a comfortable position that allows easier visualization of the entire UL. Although APWA highlights the axillary region that contains lymph nodes and main lymphatic chains, the flexion of the elbow hindered the visualization of lymphedema in this joint. In addition, participants with larger ULs may present more difficulty in the APWA position because they need sustained muscle activation to maintain their posture, potentially changing the skin temperature of the UL. Therefore, AAP is the best position for IRT assessment in BCRL diagnosis.

The maximum temperature recorded in the AAP also achieved the highest AUC, indicating moderate accuracy when compared to other temperatures in the same and other positions. This variable is more sensitive to detecting the differences between skin temperatures in ROIs than mean and minimum temperatures, and it is highly recommended in IRT for detecting vascular changes and signs of inflammation [22]. Thus, the maximum temperature is the recommended IRT measure for BCRL diagnosis.

The asymmetric pattern of ΔT between ULs serves as a functional indicator of the pathological changes [23,24]. In this study, ΔT was more accurate for assessing BCRL than the only absolute skin temperature value of the UL with lymphedema. These findings reinforce the use of IRT, consistent with previous studies that recommend recording the maximum ΔT between affected and unaffected areas for diagnostic purposes [16].

In the present study, the ULWL group exhibits cooler lower temperatures, with a ΔT of −0.45 °C identified as a suggestive cut-off point for the presence of lymphedema. Applying cut-off points suggested by the literature revealed that the closer the ΔT is to −1 °C, the higher the sensitivity and specificity for lymphedema diagnosis. Previous studies also identified lower temperatures in the UL affected by BCRL than in contralateral UL, highlighting a distinctive thermal behavior of this type of lymphedema [25]. In contrast, another study reported an increased temperature in BCRL, indicating that thermal manifestation may vary depending on the stage of lymphedema or individual characteristics [10]. Therefore, besides the use of ΔT as the most recommended measure to analyze the skin temperature of UL, the cut-off point of −0.45 °C should also be considered to identify chronic lymphedema.

Chronic inflammation, impaired lymphangiogenesis, and the deposition of fibroblasts and adipocytes contribute to reduced skin temperature. The increased number and size of lymphatic vessel structures indicates thermal dysfunction exacerbated by lymphatic flow deficiency [26]. This flow deficiency leads to the dysregulation of the autonomic nervous system, limiting efficient thermoregulation. Thus, the influence of autonomic dysfunction, mediated by the hypothalamus, highlights the critical interconnection between lymphatic flow inefficiency and autonomic regulation for heat exchange [27,28]. In this context, IRT emerges as a beneficial technique for assessing tissue thermal changes and their pathological implications in BCRL management.

Based on this study, IRT demonstrated a sensitivity of 85% and a specificity of 56% in its best configuration. Although IRT has not achieved the sensitivity and specificity observed by lymphoscintigraphy (96% and 100%, respectively) [29], this technique was considered useful and should help the clinical decision-making in BCRL. Therefore, IRT enhances, rather than replaces, the clinical assessment of BCRL. Technological advancements (e.g., improved infrared and hyperspectral imaging sensors and the development of artificial intelligence-based image processing algorithms) seek to increase the sensitivity and specificity of current IRT. Integrating these technologies into data analysis could increase the diagnostic accuracy of IRT, allowing for the more precise detection of temperature variations associated with lymphedema [30].

Environmental conditions and variations in instructions made to participants may hinder the reproducibility of IRT images. In this study, room temperature and humidity were controlled to minimize variations in image acquisition and reduce measurement bias. However, external control is not always possible in clinical practice. Therefore, ΔT may be a more pragmatic and accurate measure for BRCL diagnosis. In IRT, infrared radiation is obtained from the outer layer of the skin and tissue metabolism, providing insights into the overall functioning of the UL. The dermatological assessment guaranteed that the variations in IRT were attributable to BCRL rather than other skin conditions. In addition, skin emissivity was considered constant (ε = 0.98) in the study to ensure the accuracy of IRT measurements, following a validated standard for skin edema [10,31]. Therefore, variations in skin color did not affect this emissivity, avoiding bias in the results.

In the present study, the accuracy of IRT was compared with indirect volumetry. Although rater-dependent, this assessment is often used in clinical practice. Previous studies also highlighted the potential of IRT in BCRL diagnosis when compared to the invasive and highly precise method of fluorescence with an injection of green indocyanine [3,32]. These findings reinforce the importance of considering IRT in BCRL diagnosis as a non-invasive, painless, easily accessible, and low-cost option that does not require physical contact.

The recording and comparing of skin temperatures takes less than five minutes and provides immediate feedback, which is useful for investigating BCRL. Real-time IRT imaging may contribute to a better perception of care by participants and an understanding of their condition and clinical progress. In this sense, IRT has the potential to improve quality of life by facilitating early and less stressful BCRL diagnosis and providing health education opportunities. However, this study did not directly assess this aspect, underscoring the importance of future research to investigate the perception of participants regarding IRT. Variables, such as comfort, privacy, procedure-related anxiety, and the impact on emotional well-being and quality of life, should also be addressed.

The integration of IRT into BCRL treatment protocols requires the consideration of existing health policies, which may vary between different healthcare systems and regions. Potential barriers include the lack of established clinical guidelines for IRT use, resistance to adopting new technologies, and the need for robust evidence supporting its effectiveness and cost-effectiveness. Collaboration among researchers, clinicians, and authorities is essential to overcome these barriers and facilitate the adoption of IRT as a valuable point-of-care tool in BCRL. IRT implementation may offer significant public health benefits, especially for the early diagnosis and monitoring of BCRL [33]. However, equipment costs, the need for specialized training for healthcare professionals, and exam accessibility in less developed regions must be carefully evaluated. Cost-effectiveness studies are needed to determine the true value of IRT in the broader context of public health and BCRL management.

In this study, the restricted sample size constrained specific analyses correlating measured temperature with lymphedema stage and precluded the creation of multivariate models including different confounding variables (e.g., age, time since lymphedema diagnosis, cancer-related treatments, physical activity, diet, and metabolism) that may affect thermoregulation and temperature measurements. A total of 44 participants were selected based on specific inclusion and exclusion criteria, ensuring the analysis of 88 ULs and adequate statistical power (89%). However, the extrapolation of the results to the general population of BCRL may be limited by variations in lymphedema severity and differences in prior treatment. Another limitation concerns the heterogeneity of lymphedema distribution in the UL, which may vary among patients. Future studies should investigate the relevance of more detailed analyses by UL segments. Additionally, higher-resolution cameras could potentially provide more precise detection of subtle thermal variations, especially in the early stages of lymphedema.

Despite the recommendation to fast and avoid physical activity two hours before the examination, the type of physical activity was not controlled. Future studies should attempt to control and standardize this variable. Larger and more heterogeneous samples are essential to validate the applicability of the findings on a broader scale. Longitudinal studies are important to assess the impact of physiotherapeutic approaches on the thermal pattern of lymphedema, monitoring changes in IRT during therapy. Furthermore, future research should incorporate assessments of participant perception and comfort with the use of IRT in diagnosis.

## 5. Conclusions

IRT emerges as a complementary technique in BCRL diagnosis, offering reproducibility, reliability with moderate accuracy, and ease of use when following assessment recommendations. Image capture in the AAP is endorsed to obtain the maximum ΔT measurement. A ΔT of −0.45 °C may be considered a suggestive cut-off point for chronic lymphedema, complementing predefined cut-off points from the literature indicating abnormality (−0.3 °C to −1.0 °C).

Therefore, this study highlights the importance of IRT as a valuable complementary technique in BCRL diagnosis. Future studies should investigate the distribution of skin temperature in different stages and the severity of lymphedema, as well as its application in a broader spectrum of populations.

## Figures and Tables

**Table 1:** Sociodemographic and clinical characteristics of participants (*n* = 44). The data presented are essential for understanding the characteristics of the sample and ensuring the analysis of the representativeness and applicability of the findings.

Variables	Mean (SD) or *n* (%)
Age (years)	54 (7.57)
Time since cancer diagnosis (months)	82.88 (75.24)
Time since mastectomy (months)	78.83 (76.06)
Body mass index (kg/m^2^)	27.65 (4.80)
Associated therapies	
Chemotherapy	41 (91.1%)
Radiotherapy	40 (88.9%)
Hormone therapy	20 (44.4%)
Dominant side	
Right	38 (84.4%)
Left	6 (13.3%)
Affected upper limb	
Right	25 (55.6%)
Left	19 (42.2%)
Presence of lymphedema	26 (59.1%)

SD: standard deviation; Kg: kilogram; m^2^: square meters.

**Table 2:** Reliability of infrared thermography measurements, illustrating maximum, mean, and minimum temperature in upper limbs with lymphedema (*n* = 26) and without lymphedema (*n* = 62) groups. The table includes values of intraclass correlation coefficients, standard error of measurement, and minimal detectable change, demonstrating the accuracy and consistency of intra- and inter-rater measurements.

	ULWL					ULC				
Intra-Rater	1° Rater A Mean (SD)	2° Rater A Mean (SD)	ICC (95% CI)	SEM	MDC	1° Rater A Mean (SD)	2° Rater A Mean (SD)	ICC (95% CI)	SEM	MDC
AAP										
Tmax	31.89 (1.72)	31.92 (1.74)	1.00 (0.99–1.00)	0.00	0.01	32.42 (1.35)	32.47 (1.35)	0.99 (0.99–0.99)	0.03	0.05
Tmean	29.97 (1.47)	30.03 (1.47)	0.99 (0.99–1.00)	0.02	0.03	30.40 (1.27)	30.47 (1.29)	0.99 (0.99–0.99)	0.02	0.11
Tmin	28.15 (1.42)	28.16 (1.42)	1.00 (0.99–1.00)	0.01	0.01	28.47 (1.35)	28.52 (1.37)	0.99 (0.98–0.99)	0.03	0.09
PAP										
Tmax	30.84 (0.89)	30.80 (0.87)	1.00 (0.99–1.00)	0.01	0.02	31.29 (1.00)	31.29 (0.98)	0.97 (0.96–0.99)	0.04	0.05
Tmean	29.21 (0.94)	29.22 (0.94)	0.99 (0.99–1.00)	0.00	0.00	29.73 (1.02)	29.72 (1.02)	0.99 (0.98–0.99)	0.02	0.04
Tmin	27.73 (0.86)	27.80 (0.93)	0.99 (0.98–0.99)	0.02	0.04	28.07 (1.02)	28.05 (1.02)	0.99 (0.98–0.99)	0.02	0.04
APWA										
Tmax	32.83 (1.11)	32.91 (1.10)	0.99 (0.98–0.99)	0.00	0.00	33.16 (1.08)	33.22 (1.10)	0.99 (0.98–0.99)	0.00	0.00
Tmean	30.44 (0.91)	30.51 (0.92)	0.99 (0.99–0.99)	0.03	0.09	30.67 (1.00)	30.71 (1.01)	0.99 (0.98–0.99)	0.01	0.02
Tmin	28.59 (1.09)	28.66 (1.09)	0.99 (0.98–0.99)	0.01	0.01	28.59 (0.87)	28.61 (0.88)	0.99 (0.99–0.99)	0.01	0.01
PPWA										
Tmax	31.17 (1.01)	31.19 (1.01)	0.99 (0.98–0.99)	0.01	0.01	31.19 (0.96)	31.19 (0.96)	0.99 (0.98–0.99)	0.03	0.08
Tmean	29.37 (1.06)	29.34 (1.06)	0.99 (0.99–0.99)	0.01	0.01	29.69 (1.01)	29.68 (1.01)	0.99 (0.99–0.99)	0.01	0.01
Tmin	27.79 (0.90)	27.79 (0.90)	0.99 (0.98–0.99)	0.01	0.01	27.99 (0.91)	27.99 (0.91)	0.99 (0.98–0.99)	0.01	0.01
	**ULWL**					**ULC**				
**Inter-Rater**	**2° Rater A** **Mean (SD)**	**Rater B** **Mean (SD)**	**ICC (95% CI)**	**SEM**	**MDC**	**2° Rater A** **Mean (SD)**	**Rater B** **Mean (SD)**	**ICC (95% CI)**	**SEM**	**MDC**
AAP										
Tmax	31.92 (1.74)	31.98 (1.73)	0.99 (0.98–0.99)	0.00	0.01	32.47 (1.35)	32.54 (1.36)	0.99 (0.98–0.99)	0.00	0.00
Tmean	30.03 (1.47)	30.09 (1.45)	0.99 (0.99–0.99)	0.01	0.03	30.47 (1.29)	30.54 (1.30)	0.99 (0.99–0.99)	0.01	0.02
Tmin	28.16 (1.42)	28.25 (1.42)	0.99 (0.98–0.99)	0.00	0.01	28.52 (1.37)	28.61 (1.39)	0.99 (0.98–0.99)	0.00	0.01
PAP										
Tmax	30.80 (0.87)	3.80 (0.85)	0.99 (0.98–0.99)	0.02	0.04	31.29 (0.98)	31.29 (1.02)	0.99 (0.98–0.99)	0.02	0.04
Tmean	29.22 (0.94)	29.21 (0.94)	0.99 (0.99–0.99)	0.00	0.00	29.72 (1.02)	29.71 (1.03)	0.99 (0.99–0.99)	0.00	0.00
Tmin	27.80 (0.93)	27.70 (0.86)	0.97 (0.96–0.99)	0.07	0.21	28.05 (1.02)	28.08 (1.02)	0.99 (0.98–0.99)	0.02	0.04
APWA										
Tmax	32.91 (1.10)	32.81 (1.16)	0.99 (0.99–0.99)	0.06	0.00	33.22 (1.10)	33.20 (1.12)	0.99 (0.99–0.99)	0.03	0.09
Tmean	30.51 (0.92)	30.46 (0.98)	0.99 (0.99–1.00)	0.00	0.01	30.71 (1.01)	30.70 (1.01)	1.00 (0.99–1.00)	0.00	0.01
Tmin	28.66 (1.09)	28.57 (1.13)	0.96 (0.95–0.98)	0.09	0.11	28.61 (0.88)	28.61 (0.90)	0.97 (0.95–0.99)	0.06	0.08
PPWA										
Tmax	31.19 (1.01)	31.19 (1.08)	0.98 (0.98–0.99)	0.07	0.09	31.19 (0.96)	31.23 (1.04)	0.99 (0.98–0.99)	0.03	0.03
Tmean	29.34 (1.06)	29.35 (1.11)	0.93 (0.92–0.97)	0.05	0.08	29.68 (1.01)	29.63 (1.04)	0.97 (0.96–0.99)	0.01	0.04
Tmin	27.79 (0.90)	27.72 (0.93)	0.94 (0.95–0.98)	0.11	0.03	27.99 (0.91)	27.95 (0.92)	0.99 (0.98–0.99)	0.07	0.08

AAP: anterior anatomical position; PAP: posterior anatomical position; APWA: anterior position with arms abduction; PPWA: posterior position with arms abduction; Tmax: maximum temperature; Tmean: mean temperature; Tmin: minimum temperature; ULWL: upper limb with lymphedema; ULC: upper limb without lymphedema; SD: standard deviation; ICC: intraclass correlation coefficient; 95% CI: 95% confidence interval; SEM: standard error of measurement; MDC: minimal detectable change.

**Table 3:** Diagnostic analysis of infrared thermography for detecting breast cancer-related lymphedema. The table shows the area under the receiver-operating characteristic curve and identifies cut-off points with their corresponding sensitivity, specificity, accuracy, false positives, false negatives, true positives, and true negatives for the skin temperature of upper limbs with and without lymphedema.

Position	AUC (95% CI)	CP (°C)	Sens. (%)	Spe. (%)	TP	TN	FP	FN	Ac
AAP									
Tmax	0.456 (0.315–0.596)	32.37	0.500	0.565	13	35	27	13	0.546
Tmean	0.458 (0.322–0.595)	30.33	0.500	0.597	13	37	25	13	0.568
Tmin	0.460 (0.334–0.585)	28.25	0.615	0.548	16	34	28	10	0.568
PAP									
Tmax	0.401 (0.271–0.531)	30.88	0.500	0.613	13	38	24	13	0.580
Tmean	0.401 (0.273–0.530)	29.34	0.500	0.629	13	39	23	13	0.591
Tmin	0.458 (0.328–0.588)	27.81	0.500	0.597	13	37	25	13	0.568
APWA									
Tmax	0.493 (0.360–0.627)	32.82	0.615	0.597	16	37	25	10	0.602
Tmean	0.493 (0.365–0.621)	30.42	0.615	0.581	16	36	26	10	0.591
Tmin	0.559 (0.429–0.689)	28.33	0.692	0.548	18	34	28	8	0.591
PPWA									
Tmax	0.489 (0.354–0.623)	30.90	0.577	0.661	15	41	21	11	0.636
Tmean	0.430 (0.297–0.564)	29.30	0.500	0.629	13	39	23	13	0.591
Tmin	0.414 (0.285–0.544)	27.74	0.500	0.597	13	37	25	13	0.568

AAP: anterior anatomical position; PAP: posterior anatomical position; APWA: anterior position with arms abduction; PPWA: posterior position with arms abduction; Tmax: maximum temperature; Tmean: mean temperature; Tmin: minimum temperature; AUC: area under the curve, CP: cut-off point; Sens.: sensitivity; Spe.: specificity; 95% CI: 95% confidence interval; TP: true positive; TN: true negative; FP: false positive; FN: false negative; Ac: accuracy.

**Table 4:** Diagnostic analysis of infrared thermography for detecting breast cancer-related lymphedema. The table shows the area under the receiver-operating characteristic curve and identifies cut-off points with their corresponding sensitivity, specificity, accuracy, false positives, false negatives, true positives, and true negatives for the temperature difference between upper limbs, based on the Youden index.

Position	AUC (95% CI)	CP (°C)	Sens. (%)	Spe. (%)	TP	TN	FP	FN	Ac
AAP									
Tmax	0.700 (0.533–0.848)	0.45	0.846	0.556	20	13	5	6	0.750
Tmean	0.559 (0.374–0.744)	0.34	0.769	0.611	20	11	7	6	0.704
Tmin	0.588 (0.402–0.773)	0.10	0.808	0.556	21	10	8	5	0.705
PAP									
Tmax	0.424 (0.251–0.597)	0.12	0.538	0.667	14	12	6	12	0.591
Tmean	0.482 (0.305–0.659)	0.16	0.577	0.611	15	11	7	11	0.591
Tmin	0.351 (0.185–0.518)	0.10	0.577	0.722	15	13	5	11	0.636
APWA									
Tmax	0.524 (0.346–0.701)	0.11	0.769	0.722	22	10	8	4	0.727
Tmean	0.637 (0.460–0.813)	−0.12	0.769	0.556	20	10	8	6	0.682
Tmin	0.595 (0.409–0.781)	−0.25	0.885	0.722	18	34	28	8	0.591
PPWA									
Tmax	0.503 (0.329–0.678)	−0.10	0.654	0.667	15	41	21	11	0.636
Tmean	0.469 (0.286–0.652)	−0.10	0.577	0.667	13	39	23	13	0.591
Tmin	0.518 (0.338–0.699)	−0.25	0.731	0.722	13	37	25	13	0.568

AAP: anterior anatomical position; PAP: posterior anatomical position; APWA: anterior position with arms abduction; PPWA: posterior position with arms abduction; Tmax: maximum temperature; Tmean: mean temperature; Tmin: minimum temperature; AUC: area under the curve; CP: cut-off point; Sens.: sensitivity; Spe.: specificity; 95% CI: 95% confidence interval; TP: true positive; TN: true negative; FP: false positive; FN: false negative; Ac: accuracy.

**Table 5:** Sensitivity, specificity, accuracy, false positives, false negatives, true positives, and true negative values of temperature difference according to pre-established cut-off points from the literature for lymphedema detection.

Position	Cut-Off Point (°C)	Sens. (%)	Spe. (%)	TP/TN	FP/FN	Ac	AUC (95% CI)
AAP							
Tmax	0.3	0.769	0.500	20/9	9/6	0.659	0.700 (0.533–0.848)
	0.6	0.923	0.833	24/15	3/2	0.886	
	1	0.962	0.944	25/17	1/1	0.955	
Tmean	0.3	0.731	0.556	19/10	8/7	0.659	0.559 (0.374–0.744)
	0.6	0.885	0.944	23/17	1/3	0.909	
	1	1.000	0.944	26/17	1/0	0.977	
Tmin	0.3	0.923	0.722	24/13	5/2	0.841	0.588 (0.402–0.773)
	0.6	0.923	0.834	24/15	3/2	0.887	
	1	1.000	0.944	26/17	1/0	0.977	
APWA							
Tmax	0.3	0.923	0.833	24/15	3/2	0.886	0.524 (0.346–0.701)
	0.6	0.923	0.889	24/16	2/2	0.909	
	1	1.000	0.944	26/17	1/0	0.977	
Tmean	0.3	0.885	0.611	23/11	7/3	0.773	0.637 (0.460–0.813)
	0.6	0.962	0.722	25/13	5/1	0.864	
	1	1.000	0.944	26/17	1/0	0.977	
Tmin	0.3	0.923	0.722	24/13	5/2	0.841	0.595 (0.409–0.781)
	0.6	1.000	0.833	26/15	3/0	0.932	
	1	1.000	1.000	26/18	0/0	1.000	

AAP: anterior anatomical position; APWA: anterior position with arms abduction; Tmax: maximum temperature; Tmean: mean temperature; Tmin: minimum temperature; AUC: area under the curve; CP: cut-off point; Sens.: Sensitivity; Spe.: specificity; 95% CI: 95% confidence interval; TP: true positive; TN: true negative; FP: false positive; FN: false negative; Ac: accuracy.

## Data Availability

Data supporting the findings of this study are available on request from the corresponding author and are not publicly available due to privacy or ethical restrictions.
